# Charge Variants Characterization of Co-Formulated Antibodies by Three-Dimensional Liquid Chromatography–Mass Spectrometry

**DOI:** 10.3390/biom14080999

**Published:** 2024-08-13

**Authors:** Xiaoqing Jin, Luna Chen, Jianlin Chu, Bingfang He

**Affiliations:** 1 College of Biotechnology and Pharmaceutical Engineering, Nanjing Tech University, Nanjing 211816, China; 6610817z2001@njtech.edu.cn (X.J.); 202261218131@njtech.edu.cn (L.C.); 2 School of Pharmaceutical Sciences, Nanjing Tech University, Nanjing 211816, China

**Keywords:** charge variants, co-formulated antibodies, characterization, 3D-LC-MS

## Abstract

Co-formulated antibodies can bring clinical benefits to patients by combining two or more antibodies in a single dosage form. However, the quality analysis of co-formulated antibodies raises additional challenges, compared to individual antibodies, due to the need for accurate analysis of multiple antibodies in one solution. It is extremely difficult to effectively separate the charge variants of the two co-formulated antibodies using one ion exchange chromatography (IEC) method because of their similar characteristics. In this study, a novel method was developed for the charge variants characterization of co-formulated antibodies using three-dimensional liquid chromatography–mass spectrometry (3D-LC-MS). Hydrophobic interaction chromatography (HIC) was used as the first dimension to separate and collect the two co-formulated antibodies. The two collections were then injected into the second-dimension IEC separately for charge variants separation and analysis. Subsequently, the separated charge variants underwent on-line desalting in the third-dimension reverse-phase chromatography (RPC) and subsequent mass spectroscopy analysis. The novel method could simultaneously provide a charge variants ratio and post-translational modification (PTM) data for the two co-formulated antibodies. Therefore, it could be used for release testing and stability studies of co-formulated antibodies, making up for the shortcomings of the existing approaches. It was the first time that charge variants of co-formulated antibodies were characterized by the 3D-LC-MS method, to the best of our knowledge.

## 1. Introduction

Because of their tremendous advantages in autoimmune diseases and cancers, therapeutic antibodies have become the fastest-growing class of pharmaceutical products [1]. Five antibodies were listed in the top 10 best-selling drugs in 2023, and Keytruda (Pembrolizumab) was the best one, with gross sales of more than USD 25 billion [2]. However, monoclonal antibodies suffer from limitations, such as targeting only one epitope [3]. A combination of two or more molecules in one drug product can bring great clinical benefits to patients with higher therapeutic efficacy and improve patient compliance by targeting two or more antigens synergistically [4,5,6]. In 2020, Phesgo (Pertuzumab and Trastuzumab with hyaluronidase) [7] was approved as a fixed-dose subcutaneous injection form, significantly reducing the administration time [8]. Recently approved co-formulated antibodies include Opdualag (Nivolumab and Relatlimab) [9] and REGEN-COV2 (Casirivimab and Imdevimab) [10], which bring better benefits to the patients than the individual antibodies.

Although co-formulated antibodies offer several advantages, the characterization of co-formulated antibodies is very challenging because of the similarities between co-formulated antibodies in one solution [11]. In fact, this is a bottleneck in the co-formulated antibodies field, and there is a lack of effective methods that allow for the separation of individual antibodies to characterize their quality attributes and measure their stability. Charge variants of antibodies can probably influence stability, immunogenicity, and potency and are taken as an important quality attribute [12,13]. Since the isoelectric points (PIs) of most antibodies fall between 8 and 9 [14], charge variants of different co-formulated antibodies exhibit similar performances in analytical methods, which results in overlapping chromatograms. Consequently, it is very difficult to effectively separate and analyze each charge variant of the two antibodies simultaneously using a single analytical method. Sharma et al. utilized ion-exchange chromatography (IEC) to successfully separate two anti-HIV-1 antibodies of co-formulated antibodies, though the separation efficiency was not good, with a low resolution of charge variants for one antibody [15]. Hutanu et al. established a new method named flow-through partial filling affinity capillary electrophoresis for co-formulated antibodies and provided comparable charge variants data with a standard CZE of a single antibody [16]. Capillary isoelectric focusing (cIEF) is an alternative method to IEC and CZE for charge variants analysis and has been applied in co-formulated antibodies [17]. The current analytical methods to characterize the charge variants of co-formulated antibodies have been discussed with some limitations [18]. It is hard to obtain a suitable analytical method with a good resolution for every charge variant of the two co-formulated antibodies, even after tedious method optimization. None of these separation methods can guarantee a non-overlapping separation of the charge variants of co-formulated antibodies. Moreover, these methods provide only charge variants ratio data and no PTM data. Therefore, it may be more promising to utilize 2D-LC- or 3D-LC-coupled MS to characterize the charge variants of co-formulated antibodies.

In this study, we reported a novel 3D-LC-MS method for the charge variant characterization of co-formulated antibodies (mixed mAb A and mAb B, with a mass ratio of 1:1). HIC was selected as the first dimension to effectively separate and collect the two co-formulated antibodies. Subsequently, the two collections were separately injected into the second-dimension IEC column to separate and obtain the ratio data of the charge variants and then were on-line desalted by the third-dimension RPC column, followed by Q-TOF MS for PTM analysis. The method can simultaneously provide both the ratio data of the charge variants and the PTM data, addressing the challenge of the charge variants characterization of co-formulated antibodies by leveraging HIC differences among antibodies.

## 2. Materials and Methods

### 2.1. Reagents

Disodium hydrogen phosphate, sodium phosphate monobasic dihydrate, acetonitrile, and formic acid were obtained from Sigma-Aldrich (Darmstadt, Germany). Sodium chloride was obtained from Sigma-Aldrich (Seelze, Germany). Ammonium sulfate was obtained from Sigma-Aldrich (Missouri, MO, USA). Two mAbs (mAb A and mAb B with ≥98% SEC purity) used in this study were from our lab.

### 2.2. Instrument

The 3D-LC-MS system included a Waters H-Class (Singapore), an Agilent 1290 Infinity II (Waldbronn, Germany), and a 6545XT AdvanceBio Q-TOF mass spectrometer (Singapore). The first dimension was the Waters H-Class with an ultraviolet detector and a WFM-A fraction collector. The second dimension and the third dimension were Agilent 1290 Infinity II, with the configurations shown below: the second- and third-dimension pumps, autosamplers, thermostated column compartments, and ultraviolet detectors. The interface valve between the second dimension and the third dimension was set up with 20 µL sample loops. The 6545XT AdvanceBio Q-TOF mass spectrometer was equipped with an Agilent JetStream electrospray ionization source.

The control and data analysis of the Waters H-Class were performed by Empower 3.0. The Agilent 1290 Infinity II and the 6545XT AdvanceBio Q-TOF mass spectrometer were controlled by the MassHunter software V10.0. Data processing and theoretical molecular weight confirmation were performed by Agilent MassHunter BioConfirm V10.0 and Sequence Manger (V10.0), respectively.

### 2.3. HIC Condition

The two co-formulated antibodies were separated using a Thermo Scientific (Waltham, MA, USA) MabPac HIC-20 column (4 × 250 mm, 5 μm). The column temperature was set to room temperature. The injection volume was 5 µL (5 mg/mL protein concentration), and the detected wavelength was 280 nm. The HIC mobile phase A (HIC-A) was the combination of 100 mM phosphate buffer and 1 M ammonium sulfate, with pH 6.8, and the HIC mobile phase B (HIC-B) was 100 mM phosphate buffer with pH 6.8. The flow rate was 0.8 mL/min. The elution gradient was optimized for a good separation of the two co-formulated antibodies. The final elution gradient was as follows: 0–1 min 10% HIC-B; 1–15 min 10–100% HIC-B; 15–18 min 100% HIC-B; and 18–20 min 100–10% HIC-B.

### 2.4. IEC Condition

The charge variants were separated using a Thermo Scientific ProPac WCX-10 BioLC analytical column (4 × 250 mm, 10 μm). The column temperature was set to 45 °C. The injection volume was 20 µL (4 mg/mL protein concentration), and the detected wavelength was 280 nm. The IEC mobile phase A (IEC-A) was 20 mM phosphate buffer with a pH of 6.6, and the IEC mobile phase B (IEC-B) was a combination of 20 mM phosphate buffer and 500 mM NaCl, with a pH of 6.6. The elution gradient was as follows: 0–3 min 2% IEC-B; 3–43 min 2–22% IEC-B; 43–44 min 22–100% IEC-B; 44–46 min 100% IEC-B; 46–46.01 min 100–2% IEC-B; and 46.01–55 min 2% IEC-B. The flow rate was 0.8 mL/min.

### 2.5. 3D-LC-MS Method

**Figure 1:**
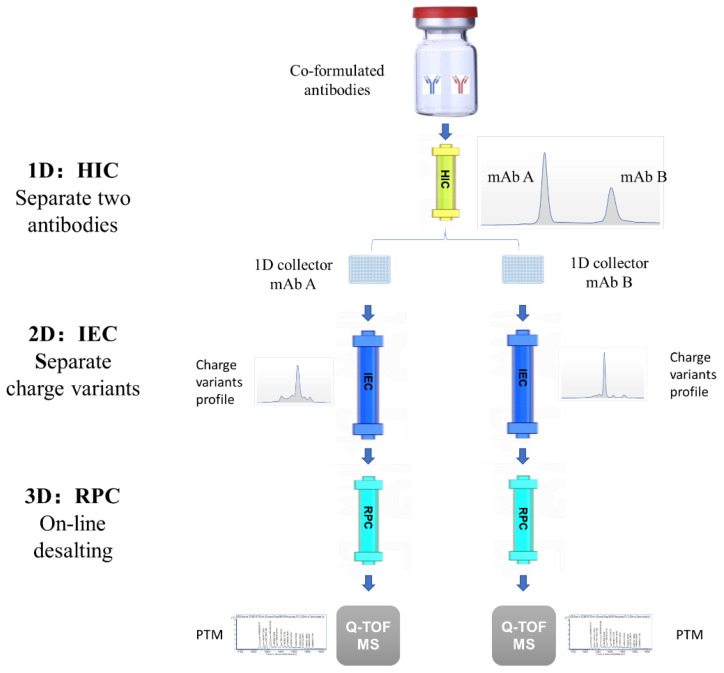
Schematic diagram of the 3D-LC-MS method for the charge variants characterization of co-formulated antibodies.

Figure 1 shows the schematic diagram of the 3D-LC-MS method for the charge variants characterization of co-formulated antibodies. The 3D-LC-MS system utilized a HIC column as the first dimension for the separation and collection of the two co-formulated antibodies, an IEC column as the second dimension for charge variants separation and analysis, an RPC column as the third dimension for on-line desalting, and a Q-TOF MS for PTM analysis.

The first-dimension HIC utilized the optimized condition shown in Section 3.1. The condition of the second-dimension IEC was the same as those mentioned in Section 2.4. Heart-cutting mode was utilized to transfer the separated charge variants in the second dimension to the third dimension. The third dimension utilized a Waters BioResolve RP mAb polyphenyl column (2.1 mm × 50 mm, 2.7 µm), and the column temperature was set to 75 °C. Mobile phase A (RPC-A) was 0.1% (*v*/*v*) formic acid in water, and mobile phase B (RPC-B) was 0.1% (*v*/*v*) formic acid in acetonitrile. The flow rate was 0.5 mL/min, and the elution gradient was as follows: 0–(cut + 10) min 5% RPC-B; (cut + 10)–(cut + 13) min 5–95% RPC-B; (cut + 13)–44.62 min 95% RPC-B; 44.62–44.80 min 95–5% RPC-B; and 44.80–55.00 min 5% RPC-B. Cut was the initial time of the peak cut. Because the retention time of each peak was different, the initial time of the peak cut was different, and the elution gradient was slightly different. The detailed gradient elution is presented in Supplementary Materials (Table S1). The PTMs of charge variants were analyzed by Q-TOF MS at the intact protein level. Positive mode was used for data acquisition at a rate of 1 Hz, with a mass range of 500–5000 *m*/*z*. The fragmentor voltage was set as 380 V.

## 3. Results

### 3.1. HIC Condition Optimization

**Figure 2:**
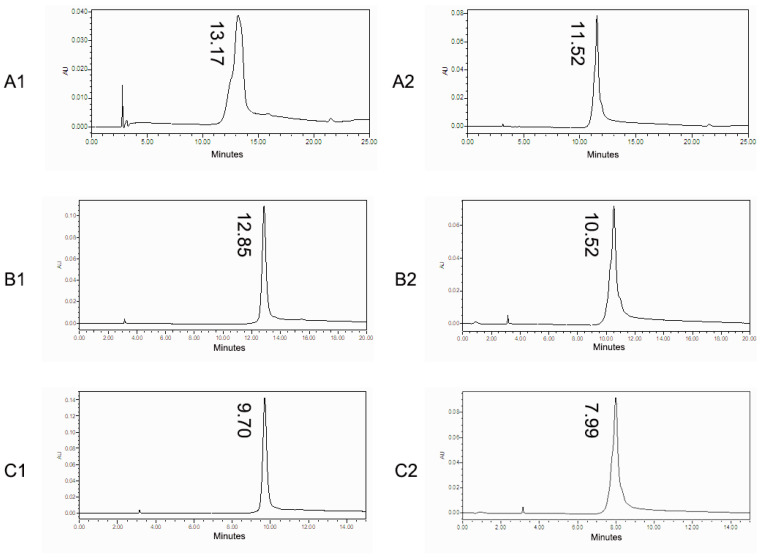
Chromatography of mAb A and mAb B in the HIC optimization. (**A1**) mAb A under the conditions of optimization 1; (**A2**) mAb B under the conditions of optimization 1; (**B1**) mAb A under the conditions of optimization 2; (**B2**) mAb B under the conditions of optimization 2; (**C1**) mAb A under the conditions of optimization 3; and (**C2**) mAb B under the conditions of optimization 3.

Three HIC elution gradient conditions were investigated in this study, and details are shown in Supplementary Materials (Tables S2–S4). The relevant chromatograms are shown in Figure 2.

Under optimization condition 1, the retention time of mAb A was 13.17 min, while the retention time of mAb B was 11.52 min, resulting in a difference of 1.65 min between them. Under optimization condition 2, the retention time of mAb A was 12.85 min, while the retention time of mAb B was 10.52 min, resulting in a difference of 2.33 min between them. Under optimization condition 3, the retention time of mAb A was 9.70 min, while the retention time of mAb B was 7.99 min, resulting in a difference of 1.71 min between them. The retention time difference between mAb A and mAb B was the biggest under the optimized condition 2. Therefore, the condition of optimization 2 was chosen as the HIC condition in the 3D-LC-MS method.

**Figure 3:**
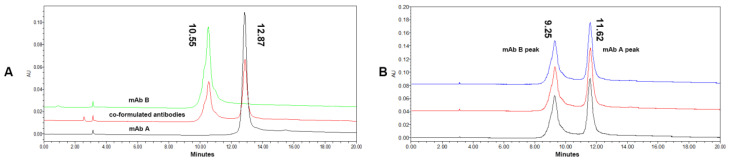
Chromatography overlay in optimized HIC. (**A**) Individual mAb A, individual mAb B, and co-formulated antibodies in optimized HIC; (**B**) co-formulated antibodies in optimized HIC in triplicate. The difference in the retention time was due to the different columns and HPLCs, but there was no detrimental effect on the resolutions of the mAb A peak and the mAb B peak.

Figure 3 shows the chromatography overlay of the individual mAb A, individual mAb B, and co-formulated antibodies under optimized HIC conditions. The resolution of the mAb A peak and the mAb B peak of co-formulated antibodies under the optimized HIC conditions was 4.11, which meant the separating effect of the mAb A peak and the mAb B peak of co-formulated antibodies was very good. Moreover, co-formulated antibodies were analyzed by the optimized HIC method in triplicate, and the results displayed good repeatability.

### 3.2. Charge Variants Characterization of Co-Formulated Antibodies by the 3D-LC-MS Method

Because the PIs and the physicochemical properties of co-formulated antibodies are similar, the retention times of charge variants are close to each other, and it is very challenging to separate all the charge variants effectively using one analytical method. Supplementary Materials Figure S1 shows the IEC chromatogram of the individual mAb A, individual mAb B, and co-formulated antibodies. The retention time range for acidic peaks of mAb B was approximately 20.0 to 23.5 min, which would cover the acidic peaks and the main peak of mAb A. The retention time range for the basic peaks of mAb A was approximately 23.5 to 27.0 min, which would cover the basic peaks and the main peak of mAb B. The number of peaks observed in co-formulated antibodies was less than the sum of the peaks from individual analyses of mAb A and mAb B, which indicated that some peaks co-eluted in IEC. Therefore, it is almost impossible to effectively separate all the charge variants of co-formulated antibodies using one IEC method.

In this study, a novel 3D-LC-MS method was developed to characterize the charge variants of co-formulated antibodies. The first dimension was HIC for the separation and collection of the two co-formulated antibodies, the second dimension was IEC for separating and analyzing the ratio of charge variants, and the third dimension was RPC for on-line desalting, followed by Q-TOF MS for the PTM analysis of charge variants.

Firstly, the 3D-LC-MS method provided the charge variants ratio data of mAb A and mAb B of co-formulated antibodies, as shown in Table 1. For mAb A of co-formulated antibodies, the average ratios of acidic peak 1, acidic peak 2, main peak 3, basic peak 4, and basic peak 5 were 11.24%, 8.58%, 69.11%, 4.79%, and 6.28%, respectively, with a low relative standard deviation (RSD) after triplicate testing. These data were very similar to the charge variants ratio data of individual mAb A using the IEC method. For mAb B of co-formulated antibodies, the average ratio of acidic peak 1, acidic peak 2, main peak 3, basic peak 4, and basic peak 5 were 14.20%, 12.15%, 49.82%, 6.69%, and 7.51%, respectively, with a low RSD after triplicate testing. These data were very similar to the charge variants ratio data of individual mAb B using the IEC method.

**Figure 4:**
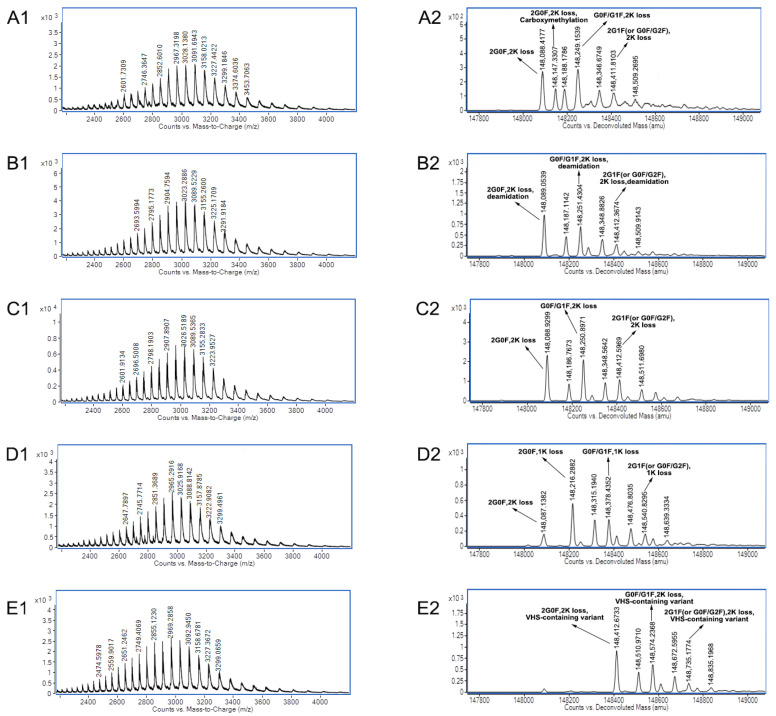
Raw MS spectra and deconvoluted MS spectra of charge variants of mAb A in co-formulated antibodies using the 3D-LC-MS method. (**A1**) Raw MS spectra of acidic peak 1; (**A2**) deconvoluted MS spectra of acidic peak 1; (**B1**) raw MS spectra of acidic peak 2; (**B2**) deconvoluted MS spectra of acidic peak 2; (**C1**) raw MS spectra of main peak 3; (**C2**) deconvoluted MS spectra of main peak 3; (**D1**) raw MS spectra of basic peak 4; (**D2**) deconvoluted MS spectra of basic peak 4; (**E1**) raw MS spectra of basic peak 5; and (**E2**) deconvoluted MS spectra of basic peak 5. There were some species with +98 Da in the deconvoluted MS spectra, which arose from the attachment of phosphoric acid. Phosphoric acid adducts were very common in MS [19] when phosphoric acid was used in the mobile phase, even though the desalting step was utilized before MS.

Secondly, the 3D-LC-MS method provided the charge variants with PTM data of mAb A and mAb B of co-formulated antibodies. Figure 4 shows the raw MS spectra and deconvoluted MS spectra of charge variants of mAb A in co-formulated antibodies using the 3D-LC-MS method.

For mAb A of co-formulated antibodies, one variant with a molecular weight of 148,088.9299 Da of main peak 3 was confirmed with the modifications 2 G0F + 2 lysine loss. Compared to the theoretical mass of 148,088.9311 Da, the mass error was only −0.0081 ppm. Two other major variants of main peak 3 were identified as variants with G0F/G1F + 2 lysine loss modifications and variants with G0F/G2F (or 2 G1F) + 2 lysine loss modifications. These glycans are usually the predominant glycans of antibodies [20]. C-terminal lysine loss in antibodies is also very common and has no effect on structure, stability, or potency, with a low risk [21]. The main MS species with 148,147.3307 Da and 148,308.1068 Da of acidic peak 1 were identified as variants with carboxymethylation modifications by mass shifts of +58.4008 Da and +57.2097 Da, which matched carboxymethylation. N-ε-carboxymethyl-lysine was one of the main products of the Maillard reaction [22]. The carboxymethylation modification of the ε-amino group on the lysine shielded the ε-amino group and appeared as an acidic peak. The main MS species of acidic peak 2 with molecular weights of 148,089.0539 Da, 148,251.4304 Da, and 148,412.3674 Da were almost the same as those of main peak 3. It was speculated that the main modification was deamidation. Deamidation is one of the main causes of acidic peaks. However, it is very challenging to distinguish such subtle mass changes (only a +0.9840 Da mass shift) at the intact protein level (~150 kDa) [23]. Compared to main peak 3, three main species of basic peak 4 had mass shifts of 127.3583 Da, 127.5381 Da, and 128.2326 Da, respectively, which matched the modification of one heavy chain with C-terminal lysine (+128 Da). As a basic amino acid, C-terminal lysine truncation, or the lack of it, will influence the charge variants. Compared to the loss of two lysines, one heavy chain with C-terminal lysine causes basic variants of antibodies [24,25]. Compared to main peak 3, three main MS species of basic peak 5 had mass shifts of 323.7434 Da, 323.3397 Da, and 322.5805 Da, respectively, which matched the modification of the signal peptide VHS (+323 Da). Usually, the signal peptide of antibodies contains VHS, and the basic peaks with untruncated signal peptide VHS were reported in papers [26,27].

**Figure 5:**
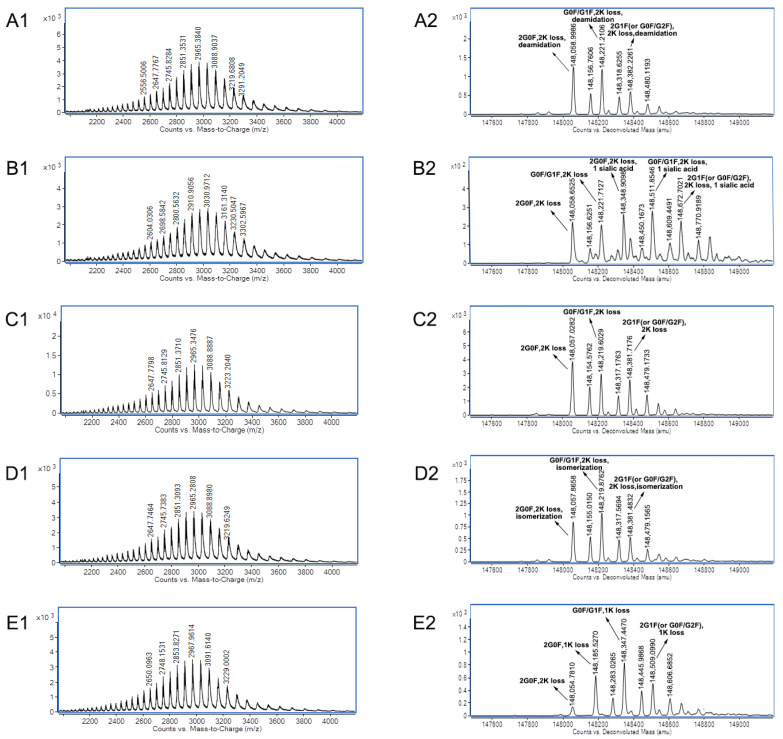
Raw MS spectra and deconvoluted MS spectra of charge variants of mAb B in co-formulated antibodies using the 3D-LC-MS method. (**A1**) Raw MS spectra of acidic peak 1; (**A2**) deconvoluted MS spectra of acidic peak 1; (**B1**) raw MS spectra of acidic peak 2; (**B2**) deconvoluted MS spectra of acidic peak 2; (**C1**) raw MS spectra of main peak 3; (**C2**) deconvoluted MS spectra of main peak 3; (**D1**) raw MS spectra of basic peak 4; (**D2**) deconvoluted MS spectra of basic peak 4; (**E1**) raw MS spectra of basic peak 5; and (**E2**) deconvoluted MS spectra of basic peak 5. There were some species with +98 Da in the deconvoluted MS spectra, which arose from the attachment of phosphoric acid. Phosphoric acid adducts were very common in MS [19] when phosphoric acid was used in the mobile phase, even though the desalting step was utilized before MS.

Figure 5 shows the raw MS spectra and the deconvoluted MS spectra of charge variants of mAb B in co-formulated antibodies using the 3D-LC-MS method. For mAb B of co-formulated antibodies, one variant with a molecular weight of 148,057.0282 Da of main peak 3 was confirmed with the modifications 2 G0F + 2 lysine loss. Compared to the theoretical mass of 148,058.8326 Da, the mass error was −12.1900 ppm. Two other major variants of main peak 3 were identified as variants with G0F/G1F + 2 lysine loss modifications and variants with G0F/G2F (or 2 G1F) + 2 lysine loss modifications. The main MS species of acidic peak 2 with the molecular weights of 148,058.9986 Da, 148,221.2106 Da, and 148,382.2261 Da, respectively, were almost the same as those of main peak 3 and were speculated to be a modification of deamidation. Compared to main peak 3, three main MS species of acidic peak 2 had mass shifts of 291.8816 Da, 292.2517 Da, and 290.9845 D, which matched the modification of glycans with one sialic acid (+291 Da). Sialic acid is one of the common glycans and one of the reasons for acidic peaks [28,29]. The main MS species of acidic peak 4 with molecular weights of 148,057.8658 Da, 148,219.8762 Da, and 148,381.4832 Da were almost the same as those of main peak 3 and were speculated to be the modification of isomerization. Isomerization of aspartic acid is a common PTM, and a greater than 45% ratio of isomerization of aspartic acid was reported [30]. Compared to main peak 3, three main MS species of basic peak 5 had mass shifts of 128.4988 Da, 127.8441 Da, and 127.3814 Da, respectively, which matched the modification of one heavy chain with C-terminal lysine (+128 Da).

Table 2 lists the PTM data summary of mAb A and mAb B of co-formulated antibodies using the 3D-LC-MS method. For mAb A of co-formulated antibodies, the main PTMs of main peak 3 were major glycans (2G0F, G0F/G2F, or 2G1F) and C- terminal lysine loss. The main PTMs of acidic peak 1, acidic peak 2, basic peak 4, and basic peak 5 were carboxymethylation, deamidation, one heavy chain with C-terminal lysine, and a VHS-containing variant. For mAb B of co-formulated antibodies, the main PTMs of main peak 3 were major glycans (2G0F, G0F/G2F, or 2G1F) and C- terminal lysine loss. The main PTMs of acidic peak 1, acidic peak 2, basic peak 4, and basic peak 5 were deamidation, glycans with one sialic acid, isomerization of aspartic acid, and one heavy chain with C-terminal lysine.

## 4. Discussion

Co-formulated antibodies are promising drug products with a higher efficacy because of the combination of different antibodies that have synergistic effects. It is a great challenge to characterize the charge variants of co-formulated antibodies due to similarities, particularly in terms of charge variants. Currently, there are two options for the testing of charge variants of co-formulated antibodies. One option is to test individual antibodies before mixing the two antibodies. It is relatively easy to test the charge variants of some antibodies using a common analytical method. However, this option has several drawbacks. The data obtained from this option do not represent the charge variants data of the co-formulated antibodies and cannot serve as release data, facing challenges from regulatory agencies. Additionally, this option is not suitable for stability studies of co-formulated antibodies. Another option is to optimize the analytical method to effectively separate and analyze charge variants of co-formulated antibodies. Nevertheless, developing an effective analytical method in practice can be highly challenging. In some cases, the charge variants of one antibody cannot be effectively separated from those of another antibody, as shown in Supplementary Materials Figure S1. In some cases, the separation result of charge variants of one antibody is not good, even though the two co-formulated antibodies can be separated effectively. Cao et al. effectively separated the two co-formulated antibodies using a long gradient elution with 85 min of the optimized IEC method, but the charge variants of one antibody had very poor peak shapes [18].

In this study, we developed a novel 3D-LC-MS method to characterize the charge variants of co-formulated antibodies. The 3D-LC-MS method utilized HIC-, IEC-, and RPC-hyphenated Q-TOF MS to characterize charge variants of co-formulated antibodies, providing not only data on the charge variants ratio but also data on PTMs. HIC was chosen as the first dimension to separate the two co-formulated antibodies because its mobile phases are relatively mild, and it is accepted as a nondestructive method [31].Therefore, the collections of HIC can be assessed for their charge variants. The retention of antibodies in the column is based on the interaction between surface amino acid residues and the HIC resin. Different antibodies have different surface amino acid residues, and this is one of the important differences among antibodies, which can be utilized to separate different antibodies. Furthermore, the mobile phase gradient of HIC has a great influence on the separation result. The elution gradient can be optimized to achieve the effective separation of different antibodies. The 2D-LC-MS (IEC- and RPC-hyphenated Q-TOF MS) is a mature technology widely used for the charge variants characterization of antibodies [32,33]. Therefore, 3D-LC-MS (HIC-, IEC-, and RPC-hyphenated Q-TOF MS) can be leveraged to establish analytical methods for the charge variant characterization of co-formulated antibodies. Considering the difference in surface amino acid residues of antibodies [34] and the powerful separation capacity of HIC [35], the 3D-LC-MS method described in this study can be used for the charge variants characterization of other co-formulated antibodies. It makes up for the shortcomings of the existing approaches and is capable of release testing and studying the stabilities of co-formulated antibodies. To the best of our knowledge, it was the first time that the 3D-LC-MS method was developed for the charge variants characterization of co-formulated antibodies. Moreover, the idea of the 3D-LC-MS method with HIC as the first dimension can address the challenge of quality analysis of co-formulated antibodies by leveraging HIC differences among antibodies, which can be used for the analysis of other quality attributes of co-formulated antibodies.

The 3D-LC-MS (HIC + IEC + RPC-hyphenated MS) was developed for the charge variants characterization of co-formulated antibodies in this study. Besides HIC, mixed-mode SEC can also be used as the first dimension for the separation of co-formulated antibodies. Mixed-mode SEC utilizes the secondary interaction (e.g., charge and hydrophobicity) during SEC separation by properly optimizing the chromatographic conditions, presenting opportunities to improve the separation of co-formulated antibodies [36]. One disadvantage is that there are less mixed-mode SEC columns commercially available than HIC columns. For the second dimension, capillary electrophoresis (cIEF, CZE, etc.) is an alternative with a high separation resolution to HPLC (IEC, etc.). MauriceFlex and CEInfinite are commercial equipment choices. However, even though new equipment emerges, such as ZipChip [37,38], the interface with MS is a bottleneck and is not used as widely as LC-MS. In the future, new combination modes can be developed for the charge variants characterization of co-formulated antibodies, such as HIC + Zipchip-coupled MS, mixed-mode SEC + IEC-hyphenated MS, mixed-mode SEC + Zipchip-coupled MS, etc.

As we know, the PTMs of co-formulated antibodies can change under stressed conditions or accelerated conditions, and these are important data in stability studies. In the future, the 3D-LC-MS method and other optimized versions can be used for the charge variants analysis of co-formulated antibodies in stability studies. This will allow the scientific community to better understand the quality change in co-formulated antibodies. On the other hand, this will help to quickly screen the stable formulation for co-formulated antibodies by comparing the PTM changes in different formulations.

## 5. Conclusions

In conclusion, we described an effective analytical method for the charge variants characterization of co-formulated antibodies using 3D-LC-MS. The first dimension utilized HIC for the separation and collection of the two co-formulated antibodies based on the difference in hydrophobic–hydrophobic interactions, the second dimension utilized IEC for analyzing and separating charge variants, and the third dimension utilized RPC for on-line desalting, followed by Q-TOF MS for the PTM analysis of charge variants. This novel method could simultaneously provide the charge variants ratio and PTM data of the two co-formulated antibodies. Because it makes up for the shortcomings of the existing approaches, this novel method could be used for release testing and stability studies of co-formulated antibodies. This innovative 3D-LC-MS method also introduces a novel approach for studying the quality attributes of co-formulated antibodies.

## Figures and Tables

**Table 1:** Charge variants ratio data of mAb A and mAb B of co-formulated antibodies using the 3D-LC-MS method.

Name	Charge Variants	3D-LC-MS Method					Data of Individual mAb A or mAb B by IEC *	RSD
		Test 1	Test 2	Test 3	Average	RSD		
mAb A	Acidic peak 1	11.06%	11.13%	11.53%	11.24%	2.26%	10.78%	2.80%
	Acidic peak 2	8.50%	8.50%	8.74%	8.58%	1.61%	8.27%	2.27%
	Main peak 3	69.22%	69.15%	68.96%	69.11%	0.19%	70.00%	0.66%
	Basic peak 4	4.80%	4.86%	4.70%	4.79%	1.59%	5.04%	2.88%
	Basic peak 5	6.42%	6.36%	6.06%	6.28%	3.11%	5.92%	3.88%
mAb B	Acidic peak 1	14.77%	14.04%	13.79%	14.20%	3.59%	14.12%	2.95%
	Acidic peak 2	12.79%	11.96%	11.71%	12.15%	4.66%	12.19%	3.81%
	Main peak 3	49.70%	50.58%	49.17%	49.82%	1.43%	50.67%	1.44%
	Basic peak 4	7.07%	6.66%	6.32%	6.69%	5.58%	7.33%	6.46%
	Basic peak 5	7.55%	7.55%	7.42%	7.51%	1.00%	7.68%	1.41%

Note: * represents the charge variants ratio of individual mAb A or individual mAb B using the IEC method.

**Table 2:** PTM data summary of mAb A and mAb B of co-formulated antibodies using the 3D-LC-MS method.

Name	Charge Variants	PTM Data
mAb A	Acidic peak 1	Carboxymethylation
	Acidic peak 2	Deamidation
	Main peak 3	C- terminal lysine loss of two heavy chains
	Basic peak 4	One heavy chain with C-terminal lysine
	Basic peak 5	VHS-containing variant
mAb B	Acidic peak 1	Deamidation
	Acidic peak 2	Glycans with one sialic acid
	Main peak 3	C- terminal lysine loss of two heavy chains
	Basic peak 4	Isomerization of aspartic acid
	Basic peak 5	One heavy chain with C-terminal lysine

Note: The PTM data of charge variants listed here are the main different modifications compared to the main peak. Since major glycans (2G0F, G0F/G2F, or 2G1F) were found in all peaks of the charge variants, major glycans were not listed as PTM data in the table.

## Data Availability

All data are contained within the article.

## Supplementary Materials

The following supporting information can be downloaded at: https://www.mdpi.com/article/10.3390/biom14080999/s1, Figure S1. IEC chromatogram of individual mAb A, individual mAb B, and co-formulated antibodies; Table S1. The elution gradient of RPC in the third dimension; Table S2. The elution gradient of HIC optimization 1; Table S3. The elution gradient of HIC optimization 2; Table S4. The elution gradient of HIC optimization 3.
