# Supplementary material for: Charge Variants Characterization of Co-Formulated Antibodies by Three-Dimensional Liquid Chromatography–Mass Spectrometry
Source: Biomolecules. 2024 Aug 13;14(8):999. doi: 10.3390/biom14080999 (PMC11352451; doi:10.3390/biom14080999)
Supplement: Supplementary file 1 [file biomolecules-14-00999-s001.zip › biomolecules-3124910-supplementary.pdf]

Supplementary Materials

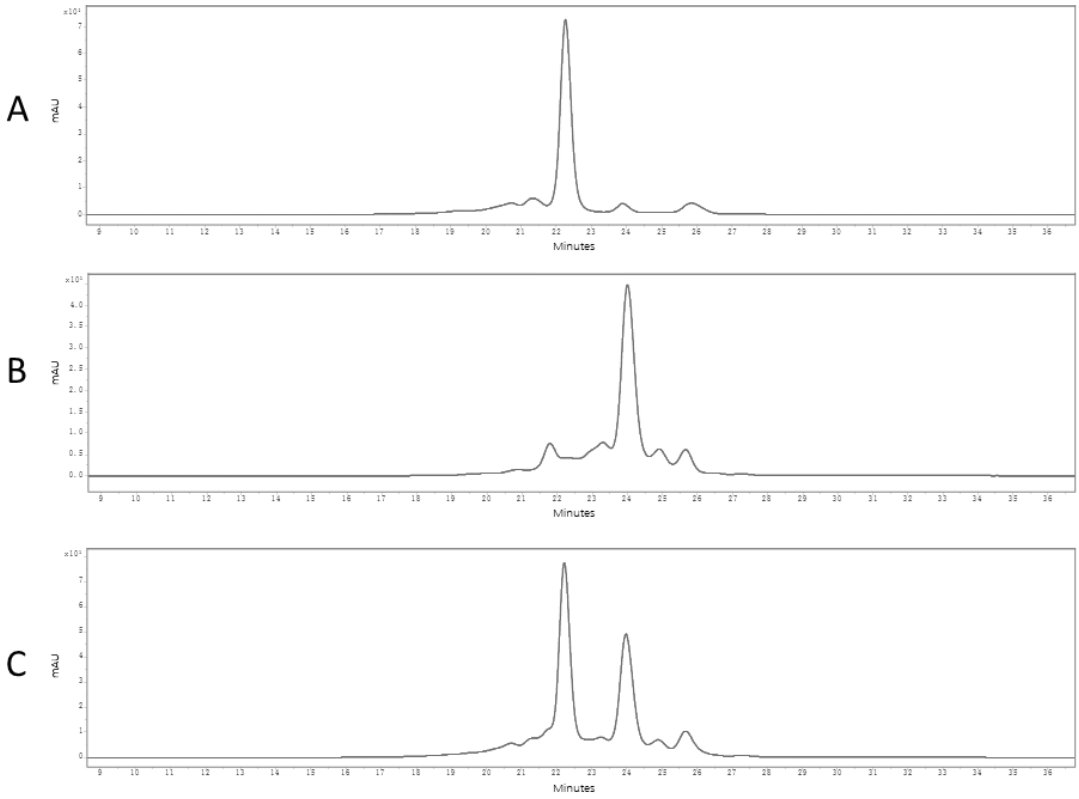

Figure S1. IEC chromatogram of individual mAb A, individual mAb B, and co-formulated antibodies. **A:** individual mAb A; **B:** individual mAb B; **C:** co-formulated antibodies

Table S1. The elution gradient of RPC in the third dimension

Time（min）	Mobile phase A（%）	Mobile phase B（%）
0.0	95	5
Cut*+10	95	5
Cut+13	5	95
44.62	5	95
44.80	95	5
55.0	95	5

Note: *Cut was the initial time of peak cut. Because the retention time of each peak was different, the initial time of peak cut was different, and the elution gradient was slightly different.

Table S2. The elution gradient of HIC optimization 1

**Time（min）**	**Mobile phase A（%）**	**Mobile phase B（%）**
0	100	0
1	100	0
15	0	100
18	0	100
20	100	0
25	100	0

Table S3. The elution gradient of HIC optimization 2

**Time（min）**	**Mobile phase A（%）**	**Mobile phase B（%）**
0	90	10
1	90	10
15	0	100
18	0	100
20	90	10

Table S4. The elution gradient of HIC optimization 3

**Time（min）**	**Mobile phase A（%）**	**Mobile phase B（%）**
0	80	20
1	80	20
10	0	100
13	0	100
15	80	20
